# Hybrid Curcumin Compounds: A New Strategy for Cancer Treatment

**DOI:** 10.3390/molecules191220839

**Published:** 2014-12-12

**Authors:** Marie-Hélène Teiten, Mario Dicato, Marc Diederich

**Affiliations:** 1 Laboratory of Molecular and Cellular Biology of Cancer (LBMCC), Hôpital Kirchberg, 9, Rue Edward Steichen, Luxembourg L-2540, Luxembourg; E-Mails: marie_helene.teiten@lbmcc.lu (M.-H.T.); mdicato@gmail.com (M.D.); 2 Department of Pharmacy, College of Pharmacy, Seoul National University, 1 Gwanak-ro, Gwanak-gu, Seoul 151-742, Korea

**Keywords:** curcumin, hybrid molecules, cancer, bioavailability

## Abstract

Cancer is a multifactorial disease that requires treatments able to target multiple intracellular components and signaling pathways. The natural compound, curcumin, was already described as a promising anticancer agent due to its multipotent properties and huge amount of molecular targets *in vitro*. Its translation to the clinic is, however, limited by its reduced solubility and bioavailability in patients. In order to overcome these pharmacokinetic deficits of curcumin, several strategies, such as the design of synthetic analogs, the combination with specific adjuvants or nano-formulations, have been developed. By taking into account the risk-benefit profile of drug combinations, as well as the knowledge about curcumin’s structure-activity relationship, a new concept for the combination of curcumin with scaffolds from different natural products or components has emerged. The concept of a hybrid curcumin molecule is based on the incorporation or combination of curcumin with specific antibodies, adjuvants or other natural products already used or not in conventional chemotherapy, in one single molecule. The high diversity of such conjugations enhances the selectivity and inherent biological activities and properties, as well as the efficacy of the parental compound, with particular emphasis on improving the efficacy of curcumin for future clinical treatments.

## 1. Introduction

Research on potential anticancer drugs started in the early 1900s [1] and rapidly included investigations on natural products. By embedding experiences from the past Ayurveda and traditional Chinese medicines [2], which were based on the principle of a multi-component therapy that involves synergistic interactions giving rise to a therapeutic effect [2,3,4,5], and by exploiting modern systems and molecular biology, pointing out the network complexity and pathway redundancy that are implicated in living systems and resistance to treatment, it appeared clear to consider multicomponent therapeutics for the treatment of complex diseases [6,7,8]. With this in mind, the drug discovery paradigm was originally based on a “one target, one disease” approach, but subsequently shifted to the “multi-target paradigm” in order to develop agents able to modulate multiple targets simultaneously [9,10]. This new concept opens new horizons for the exploration of natural sources with the perspectives of safety and efficacy, as well as the improved compliance of patients [11]. Thus, over the past 50 years, various quests for natural compounds exhibiting anticancer potential allowed the discovery of several natural compound families, such as anthracyclines (doxorubicin, daunorubicin), vinca alkaloids (vincristine, vinblastine), epipodophyllotoxin lignans, camptothecin derivatives (topotecan) and taxanes (paclitaxel), considered as the backbone of conventional chemotherapy [12]. Besides these, other compounds were isolated from the diet (fruits and vegetables), such as curcumin, resveratrol, epigallocatechin-3-gallate (EGCG) or emodin [13,14], and marine organisms (e.g., Arabinosylcytosine (Ara-C), aplidin, squalamine) [15,16] and characterized for their multi-target anticancer potential [17].

This review focuses on the multi-target dietary natural compound, curcumin, which exhibits much biological and medicinal value, but is limited in its future clinical use due to its low bioavailability. We will summarize here some standard strategies to overcome this weakness, including the design of synthetic analogs, the combination with specific adjuvants and nano-formulations. Furthermore, we will give more details about a promising approach leading to the development of hybrid curcumin molecules considered as multifunctional compounds.

## 2. Curcumin

**Figure 1:**
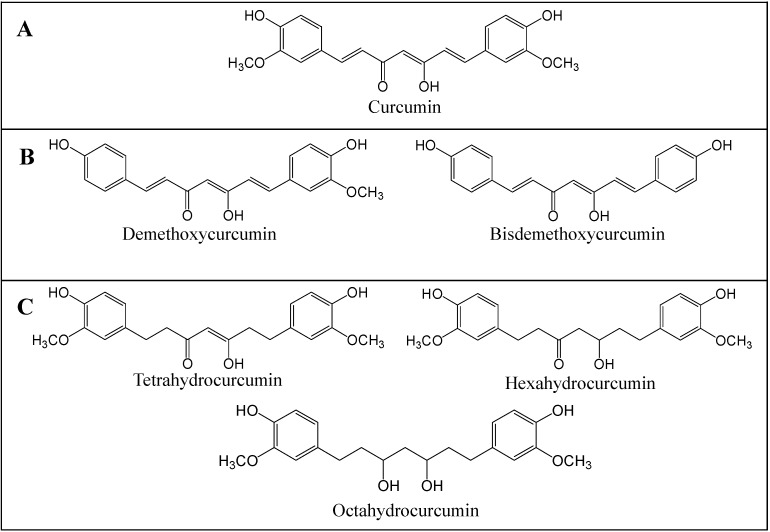
Chemical structure of curcuminoids. Curcumin (**A**); Curcumin demethoxy derivatives (demethoxycurcumin and bisdemethoxycurcumin) (**B**); Hydrogenated curcumin metabolites (tetrahydrocurcumin, hexahydrocurcumin and octahydrocurcumin) (**C**).

Curcumin or diferuloylmethane (Figure 1) is a yellow spice that is used in curry. It is extracted from the rhizome of the plant, *Curcuma longa*, and has been used for centuries in Ayurvedic, Chinese and Hindu traditional medicines as a potent anti-inflammatory agent. Research over the last 50 years established that curcumin appears both as a preventive and therapeutic agent able to reverse, inhibit or prevent the development of cancer by inhibiting specific molecular signaling pathways involved in carcinogenesis, as reported in *in vitro* [18,19,20,21,22], *in vivo* and in clinical studies [23,24,25,26]. Studies performed *in vitro* pointed out that this natural compound appears as an interesting epigenetic modulator [27,28] that possesses anti-oxidant [13,29,30], anti-inflammatory [31,32,33], anti-proliferative [34,35] and anti-angiogenic [36,37] properties in the micromolar concentration range in several cancer cell types. Curcumin’s structure-activity relationship was established by the comparison of the bioactivity of curcumin and its naturally occurring analogs, including its demethoxy derivatives (demethoxycurcumin and bisdemethoxycurcumin) and its active hydrogenated metabolites (tetrahydrocurcumin, hexahydrocurcumin and octahydrocurcumin) (Figure 1) [38,39,40], but also by the synthesis of curcumin analogs [41,42,43]. It became clear that the high anti-inflammatory and anti-tumor potentials of curcuminoids are related to their low level of hydrogenation and high level of methoxylation, but also to the high level of unsaturation of the diketone moiety [44]. The radical scavenging potential of the curcuminoids was linked to the number of *ortho*-methoxy substitutions and to the level of hydrogenation of the heptadiene moiety of curcumin [30,45]. Indeed, glycosylation of curcumin’s aromatic ring makes this compound more water soluble with a greater kinetic stability and a good therapeutic index [46].

Unfortunately, even if curcumin is well tolerated by patients and prevents the formation of xenografted tumors in rodents by acting on a variety of molecular targets involved in cancer development [47], preclinical studies revealed that oral administration of 10 to 12 g of curcumin leads to plasma concentrations of curcumin in patients that are in the nanomolar range (less than 50 nM) [47], due to its poor solubility in aqueous medium and its short biological half-life, linked to rapid metabolism and elimination by the liver [25,48]. So far, the poor pharmacodynamics of curcumin has been one of the factors that has hampered its translation into clinical applications.

## 3. Strategies to Enhance Curcumin Bioavailability

**Figure 2:**
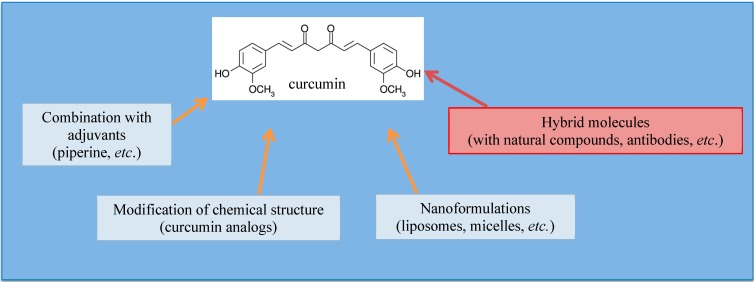
Strategies to overcome the low bioavailability and solubility of curcumin. These strategies consist of the modification of the chemical structure of curcumin, in the combination of curcumin with adjuvants, in liposomal or micellar nano-formulations of curcumin and, more recently, in the design of hybrid molecules consisting of the conjugation of curcumin with an adjuvant, antibodies or other natural compounds.

Several delivery strategies with novel curcumin formulations have been explored to overcome low oral bioavailability and so, also, research aimed at increasing the compound’s anticancer potential in patients through optimization of its absorption and serum concentration. Moreover, new formulations should also improve metabolism and provide an excellent ratio between the desirable an undesirable side effects [49,50,51]. Novel approaches included the combination with specific adjuvants and formulations in micelles, liposomes, nanoparticles or phospholipid complexes, as well as the design of synthetic analogs (Figure 2).

### 3.1. Curcumin Analogs

The structure activity relationship (SAR) of curcumin was established based on the design and the analysis of the SAR and anticancer effect of curcumin-derived molecules [38,41,42,43,52]. Structural modifications of the curcumin scaffold were also elaborated in order to improve the low bioavailability of curcumin by increasing its hydrophilicity, by facilitating its transmembrane passage and increasing the delay of metabolism. Among the curcumin analogs designed so far, dimethylcurcumin appears as a promising curcumin analog [53], as it exhibits a higher bioavailability in mice compared to the natural compound with improved apoptotic properties in colorectal cancer cells [54], as well as potent anti-inflammatory properties in both murine and human lymphocytes [55], such that it is preferentially used in clinical studies. Similarly, symmetrical 1,5-diarylpentadienone curcumin analogs, whose aromatic rings possess two alkoxy substitutes, were reported to exhibit 30-times higher growth suppressive activity than curcumin without *in vivo* toxicity. These analogs decreased the expression levels of oncoproteins, including β-catenin, Ki-Ras, cyclin D1 and ErbB-2, at concentrations much lower than those required for curcumin in HCT116 colon cancer cells [56]. Many other curcumin analogs were designed and evaluated for their impact on the nuclear factor-κB (NF-κB) signaling pathway, but their bioavailability remains yet to be established [57,58,59].

### 3.2. Combination with Specific Adjuvants

Another strategy to enhance curcumin oral bioavailability and plasma retention time consists of blocking the metabolic sites of this molecule by the use of adjuvants able to counteract detoxification enzymes implicated in curcumin metabolism.

The best described enhancer of curcumin bioavailability is piperine, a molecule isolated from black pepper [60]. This compound acts on the ultrastructure of the intestinal brush border, which leads to increased molecule absorption. Piperine is also described for its impact on cell metabolism through inhibition of UDP glucuronosyltransferases (UGTs) and cytochrome p450s, as well as for its effect on p-glycoprotein (Pgp), implicated in multidrug resistance (MDR) [61]. The concomitant administration of piperine with curcumin in animals or human was effectively reported to increase the serum concentration of curcumin by two thousand-times, due to extension of the absorption and bioavailability of curcumin with no adverse effects [62].

The use of epigallocatechin-3-gallate (EGCG) as an adjuvant to curcumin was reported to enhance curcumin bioavailability. Such a combination leads to a significant reduction of uterine leiomyosarcoma SKN cell proliferation through the inhibition of protein kinase B (PKB)/AKT, mammalian target of rapamycin (mTOR), S6 kinase (S6K) phosphorylation and through the induction of apoptosis at a much lower curcumin concentration than the one required for curcumin alone [63].

### 3.3. Curcumin Nano-Formulations

Nano-formulations [64,65,66] aim to improve the delivery of the hydrophobic curcumin molecule via liposomal, micellar or phospholipid complex formulations. Moreover, these nano-sized entities were designed and investigated to improve bioavailability and systemic delivery.

Micelles are conjugates of hydrophobic drugs and water-soluble polymers with an intrinsic cell-specific binding capacity acting as target-specific drug carriers. Drug payload usually occupies the micelle core. Several types of polymers were tested in order to improve intestinal absorption of curcumin to enhance its bioavailability. Thus, multiple curcumin molecules were conjugated to different types of polymers, such as poly(lactic) acid, via Tris and methoxy-poly(ethylene glycol) (PEG) [67,68,69,70] to the C-6 carboxylate functionality of hydrophilic sodium alginate via an ester linkage [71] or to hyaluronic acid, a naturally-occurring polysaccharide composed of *N*-acetyl-d-glucosamine and d-glucuronic acid that presents a strong affinity with cell-specific surface markers, such as CD44 [72]. In both cases, the resulting polymer-curcumin conjugate micelles were shown to enhance the aqueous solubility and stability of curcumin, as well as its intracellular delivery and subsequent cytotoxicity in hepatocellular carcinoma HepG2 cells or L-929 fibroblasts cells.

Liposomes are aggregates of hundreds of phospholipid molecules into a spherule, which compartmentalizes bioactive compounds. Such a drug delivery system can improve the therapeutic outcome by delivering the drugs to their site of action and by maintaining therapeutically relevant drug levels for prolonged treatment periods [73]. Several studies evaluated the effect of liposome-encapsulated curcumin or combinations of curcumin with conventional chemotherapeutic agents or even chemopreventive agents from dietary origins.

*In vitro* results show that liposomal curcumin induces similar effects as free curcumin on human pancreatic carcinoma cell proliferation and nuclear factor kappa-light-chain enhancer of activated B-cell (NF-κB) signaling at equimolar concentrations. Liposomal curcumin downregulated the NF-κB pathway by consistently suppressing NF-κB binding to DNA, by decreasing the expression of NF-κB-regulated genes, including cyclooxygenase-2 (COX-2) and interleukin (IL)-8, both implicated in tumor growth and invasiveness, and subsequently induced apoptosis. *In vivo* data demonstrated improved bioavailability: liposomal curcumin suppressed pancreatic carcinoma growth in murine xenograft models and inhibited tumor angiogenesis by decreasing the expression of CD31 (endothelial cell marker), vascular endothelial growth factor (VEGF) and IL-8 [74]. Improved bioavailability and bioactivity after encapsulation in liposomes were validated in other cellular models, such as MCF-7 breast cancer [75], HeLa and SiHa cervical cancer cells [76], head and neck squamous cell carcinoma (HNSCC) CAL27 and UM-SCC1 cell lines *in vitro* and *in vivo* [77].

Curcumin was then co-encapsulated in liposomes with conventional chemotherapeutic agents, such as oxaliplatin [78], or with other dietary chemopreventive agents, such as resveratrol [79]. In both instances, these formulations led to a synergistic effect in LoVo colorectal cancer cells and xenografts, as well as in prostate cancer xenografted mice with reduced cancer growth and incidence.

Nano-formulations were also used to combine curcumin with conventional anticancer drugs. Poly(d,l-lactide-co-glycolide acid) (PGLA) nanodrug formulations are gaining interest for nanomedicine applications [80], as this approach helps to overcome the lack of specificity of anticancer drug delivery and, thus, to protect neighboring normal healthy cells. With this in mind, curcumin was conjugated to 5-fluorouracil (5FU) in polymeric magnetic nanoparticles encapsulated with poly(d,l-lactide-co-glycolide) acid. This therapeutic nano-formulation exhibits a multimodal efficacy, as curcumin and 5FU act synergistically by enhancing cellular uptake and accumulation, by inducing destabilization of the cytoskeleton and loss of mitochondrial membrane potential, initiating early and late apoptosis in MCF-7 breast cancer cells [81].

Failure of treatment with the conventional chemotherapeutic drug, doxorubicin, is linked to the development of multidrug resistance mediated by Pgp. The design of poly(d,l-lactide-co-glycolide) nanoparticles combining doxorubicin and curcumin benefits both compounds. On the one hand, it improved the delivery of curcumin in K562 cancer cells, which contributes to the inhibition of the development of drug resistance against doxorubicin. The overall result is the enhancement of the antiproliferative activity of doxorubicin in K562 cells. In that case, curcumin not only facilitates the retention of doxorubicin in the nucleus for a longer period of time, but also inhibits MDR1 and BCL-2 expression and, finally, leads to apoptosis [82,83].

### 3.4. Concept of “Hybrid Molecules”

**Figure 3:**
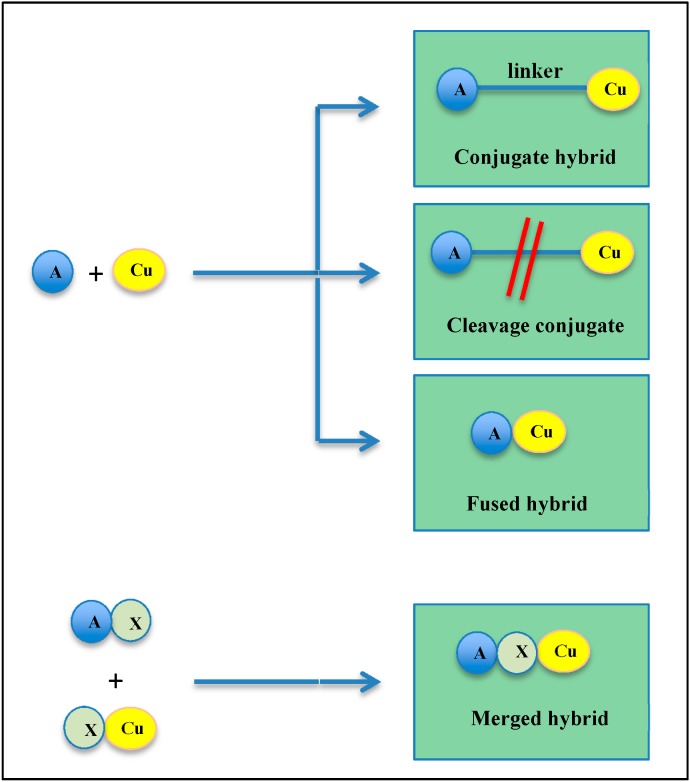
Different approaches for the design of hybrid molecules. The linkers used to connect two pharmacophores can vary and allow defining different types of hybrid molecules: conjugate hybrid, cleavage hybrid, fused hybrid and merged hybrid. (Cu, curcumin; A, molecule; X, common pharmacophore).

The “hybrid molecules” concept emerged from combination therapies, consisting of the administration of a cocktail of drugs, traditionally used by clinicians to treat unresponsive patients [84,85,86]. The multicomponent therapeutic strategy aims at combining two drugs acting by different mechanisms. Again, the aims are reduced side effects when used at their optimal dose, the design of chemical entities with improved efficacy and triggering less resistance [2,6,8]. By taking into account the knowledge about the pharmacological, structural and molecular interaction profiles of anticancer drugs [7,87,88], hybrid molecules, also called multifunctional or conjugated drugs, are designed by chemical hybridization, wherein two or more drugs having different activities can be co-formulated by connection through a stable or metabolizable linker, allowing simultaneous delivery [6,89,90]. The overall strategy is adopted to allow synergy and to improve the pharmacokinetic and pharmacodynamic profiles of the combined compounds, so that each component of the hybrid counterbalances the other’s side effects. This approach allows one to improve drug bioavailability and transport across membranes of cell organelles, but also to protect active substances from enzymatic degradation. By using hybrid molecules, the risk of drug-drug interactions can be minimized and potential drug resistance avoided [4,91,92,93,94]. Depending on their linker, hybrid molecules are classified into four groups (Figure 3): (i) “conjugates” in which hybrids are composed of pharmacophores that are separated by a distinct metabolically-stable linker group that is found in either of the individual drugs; (ii) “cleavage conjugates” present a linker designed to be metabolized, in order to allow the release of the two drugs, which interact independently with each target; (iii) the size of the linker can be decreased until the scaffolds are touching, so that hybrids appear as “fused” hybrid molecules; (iv) however, hybrids are most commonly designed as “merged hybrids”, where scaffolds are merged by taking advantage of the common structural features of the starting compounds to generate smaller and simpler molecules [9,95].

Mother Nature is a huge source of natural compounds providing scaffolds that can be combined, such that potent hybrids emerge as a novel drug discovery approach. Such hybrid molecules based on natural products can be generated naturally or synthetically, by combining entire or partial natural scaffolds [96,97]. Resulting hybrids can present similar or different mechanisms of action or can even target specific organs [89]. Natural product hybrids, comprising chalcones, coumarins, stilbenes and other scaffolds, were mainly applied in drug discovery against cancer and neurodegenerative disorder (Alzheimer’s disease, AD) [98,99,100,101,102,103,104,105]. Selected molecules were already reported in a number of U.S. patents [91]. Among them, a patent filed in 2011 highlighted that the hybrid, combining the two dietary compounds, curcumin and β-ionone, can be used as a bifunctional antiandrogen and multi-targeting agent in both hormone-sensitive (LNCAP) and hormone-independent (22Rv1) prostate cancer cells by inhibiting androgen receptor (AR) signaling and IκB kinases [106]. Such hybrids present also potent dose-dependent cytotoxic activities in circumventing resistance to the current antiandrogens used in clinics. More recently, methylated curcumin-resveratrol hybrid molecules, which improve curcumin bioavailability and bioactivity, were also approved by U.S. patent for the treatment of cancer [107]. Such curcumin-resveratrol hybrids are also under investigation for approval by the European patent office as a novel drug for the treatment of Alzheimer’s disease [108].

Patent application and approval of curcumin hybrid molecules, as well as the well-described bioactivity of curcumin underline the importance of such formulations in the field of drug discovery for the treatment of incurable diseases. We will give hereafter an overview of the different formulations of curcumin as hybrids or conjugates, their impact on curcumin bioavailability, as well as their multifunctional properties on cancer, neurodegenerative disorder and HIV.

## 4. Multifunctional Curcumin Molecules in the War against Cancer

### 4.1. Curcumin Conjugated with Piperine and Amino Acids

Based on previous studies, it was highlighted that the combination of piperine (1-piperoyl piperidine) and curcumin enhances bioavailability by reducing the rapid metabolism in the liver and intestinal wall. It was published that the inhibition of hepatic and intestinal glucuronidation, both in rats and humans, was observed, without adverse effects [62]. Moreover, bioconjugates, associating curcumin with ligands that further target internalization, were designed and described.

**Figure 4:**
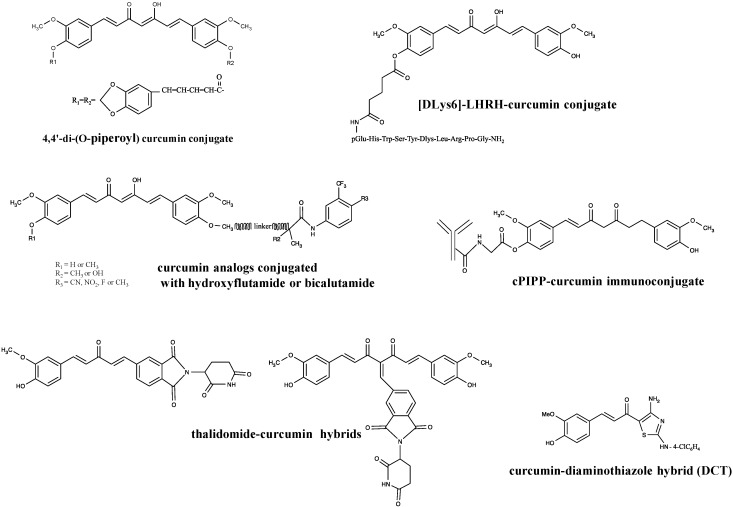
Chemical structure of curcumin hybrid compounds with anticancer properties. LHRH, luteinizing hormone releasing hormone.

Piperine and glycine appeared as the most widely used curcumin ligands, as glycine attenuates the increase of intracellular calcium. With this in mind, Mishra *et al.* synthetized different curcumin derivatives conjugated to glycine and piperic acid, designed as 4,4'-di-(O-glycinoyl) curcumin and 4,4'-di-(O-piperoyl) curcumin (Figure 4), and tested them for their differential and redox regulatory activities in AK-5 rat histiocytoma cells. They pointed out that these dipiperoyl and diglycinoyl derivatives present high apoptotic activity through the downregulation of Bcl-2 and cleavage of pro-caspase-3 at low concentrations and that this downregulation correlates with the generation of ROS without altering GSH levels [109]. The same curcumin conjugates were tested for their impact on the nuclear E6 protein of human papillomavirus type 16 (HPV-16 E6), known to target p53 and pRb tumor suppressors for ubiquitin degradation, as HPV is the major protein participating actively in the development of oral and cervical cancers [110]. Results obtained by docking analysis revealed that curcumin and its conjugate bind to different active sites on HPV-16 E6 protein, which represent ideal targets for restoring the tumor suppressor function of p53 and, thus, allowing the apoptosis of infected cells. However, in this instance, the curcumin-piperoyl conjugate is less effective than curcumin alone for HPV-16 inhibition [111]. Improved results on HPV-16 could be expected *in vitro* and *in vivo* with the 4,4'-O-dipiperoyl ester of curcumin by taking into account its well-described anticancer potential observed in AK-5 rat histiocytoma cells.

In another approach, curcumin was also conjugated with glycine, glutamic acid, valine and demethylenated piperic acid. These amino acids were used as carrier proteins that prevent the metabolic degradation of curcumin. The anticancer properties of these curcumin bioconjugates were assessed in HeLa and KB cells. Among all of the ligands selected for their capacity to enhance curcumin bioavailability, the curcumin glutamoyl derivative appeared to be the most effective compound when tested for antiproliferative potential. Demethylenated piperic acid, in which the methylenedioxy ring was opened, appeared as the most active compared to the curcumin-piperic acid conjugate. The generation of free radicals and subsequent impairment of the cellular anti-oxidant defense were described as early events leading to cell death by apoptosis of the tested cancer cell models treated with these conjugates [112].

Conjugates of curcumin with piperic acid (CDP) were synthetized by esterifying the 4 and 4' phenolic hydroxyls, which are the metabolic sites for sulfation and glucuronidation, in order to delay the metabolic rate of degradation of curcumin and subsequently enhance its bioavailability. Piperine helps to overcome the efflux of the hydrophobic curcumin molecule by multidrug resistance Pgp. As for the natural curcumin molecule, di-O-piperoyl curcumin (CDP) and di-O-glycinoyl curcumin (CDG) conjugate were shown to present a high anticancer potential *in vitro* in MCF-7 and MDA-MB-231 breast cancer cells, in which they decrease cell viability and induce changes of nuclear morphology in a micromolar concentration range. Moreover, CDP was described to induce cell death by the production of ROS via the mitochondrial apoptotic pathway through permeabilization of the mitochondrial membrane, leading to the release of cytochrome c, apoptosis inducing factor (AIF), small-molecule second mitochondria-derived activator of caspases (Smac) and other apoptogenic proteins, such as Bcl-2 and Bcl-xL, through the inhibition of nuclear translocation of transcription factor NF-κB and, finally, through chromatin condensation and fragmentation. In conclusion, this designed conjugate of curcumin does not affect the anti-tumor efficacy of the natural compound, while it enhances its bioavailability by modulating efflux mechanisms. Such results promise an enhanced pharmacokinetic profile for further *in vivo* applications [113].

Kumar *et al.* published molecular docking studies related to the effect of curcumin conjugated to amino acids on the activation of the signal transducer and activator of transcription (STAT)3 transcription factor, known to promote the expression of genes involved in cell growth, proliferation and survival [114]. They pointed out that residues, including LYS-591, ARG-609, SER-611, GLU-612, SER-613 and SER-636 VAL-637, should play an important role in the binding of curcumin-amino acid conjugates with the Src homology (SH2) domain of the STAT3a monomer. Moreover, the authors showed that the curcumin-proline conjugate (1,7-bis(4-*O*-l-prolinoyl-3-methoxyphenyl)-1,4,6-heptatriene-5-ol-3-one) was the most potent inhibitor of STAT3 dimerization among all conjugates tested [115].

### 4.2. [DLys6]-LHRH-Curcumin Conjugate

Gonadotropin-releasing hormone (GnRH), also called luteinizing hormone releasing hormone (LHRH), and its relative receptor (GnRHR, LHRHR) are overexpressed in different types of cancer and present a limited expression in normal tissues. They take part in the autocrine/paracrine regulatory system of cell proliferation of several human malignant solid tumors, such as pancreatic or gynecological cancers [116,117,118,119]. GnRH is a neurohormone that stimulates the synthesis and secretion of the gonadotropins, follicle-stimulating hormone (FSH) and luteinizing hormone (LH). GnRHRs are generally characterized in terms of binding affinity for GnRH analogs, which allows insight into the role of GnRHR in tumor growth, progression and vascularization [117]. Their activation is known to trigger strong antitumor activity. This opens the way to the search for GnRH analogs able to target these GnRHR receptors in different tumor cell models and, subsequently, to specifically deliver anticancer drugs to tumors. Novel therapeutic strategies consisting of the design of specific hybrids, in which a highly-specific GnRH analog ([DLys6]GnRH) was linked to specific cytotoxic compounds traditionally used in clinics (cisplatin, doxorubicin), to phytochemicals well characterized for their anticancer properties (curcumin) or linked to nanocarrier-based delivery system, were thus developed [117].

Curcumin was conjugated to the synthetic GnRH agonist, [DLys^6^]-LHRH. The effect of this hybrid was evaluated on MIAPaCa-2, BxPC-3 and Panc-1 pancreatic cancer cells *in vitro* and *in vivo*. This hybrid was shown to inhibit pancreatic cancer cell proliferation and to induce apoptotic cell death mediated by caspase-3 and PARP (poly(ADP-ribose) polymerase) cleavage with an equal efficacy to free curcumin at equimolar concentrations *in vitro*. Interestingly, this conjugate presents improved water solubility compared to free curcumin, which allows its intravenous administration. Therefore, this approach could be interesting for future *in vivo* applications and translation into clinical use.

The [DLys^6^]-LHRH-curcumin conjugate (Figure 4) was then reported to prevent the growth of MIAPaCa-2 pancreatic cancer cell xenografts in nude mice compared to free curcumin, free [DLys^6^]-LHRH or vehicle used alone. In conclusion, by its specific targeting of tumor cells, its solubility and its impact on cancer cell proliferation *in vitro* and *in vivo*, the [DLys^6^]-LHRH-curcumin hybrid appears attractive for further investigations and opens the way for the development of other GnRH analog-based nutraceutic hybrids [120].

### 4.3. Curcumin Conjugates with Antiandrogens

Curcumin was also conjugated to clinically used antiandrogens, such as flutamide and bicalutamide, in order to mitigate the side effects of these drugs while inhibiting the proliferation of androgen-dependent (LNCaP) and -independent (PC-3) prostate cancer cell models (Figure 4). Both conjugates demonstrated more potent antiproliferative effects than the tested antiandrogen alone and strongly inhibited actin-based pseudopodia formation, known to be highly implicated in cell migration and tumor metastasis. The mechanisms of action of curcumin and curcumin conjugates are linked to their intracellular distribution. Curcumin, which mainly accumulates in cell nuclei, inhibits cell cycle progression by targeting the functional proteins in the nuclear region, whereas its conjugates, which mainly localize in the cytosol, induce irregular nuclear division, which leads to cell death by apoptosis [121].

### 4.4. Curcumin Immunoconjugates

The use of recombinant antibodies for cancer therapy and diagnosis is a well-accepted strategy. Most antibodies are designed to target cytokines (like TNFα) or growth factors (like vascular endothelial growth factor, VEGF) in order to disrupt corresponding tumorigenic molecular pathways [122,123,124]. Recent approaches associate such antibodies with natural compounds to further improve the biomolecular activities of natural compounds by selectively targeting them to cancer cells.

In the complex field of cancers expressing ectopically human chronic gonadotropin (hCGβ), poor prognosis and adverse survival are encountered [125], so that research teams engineered a recombinant chimeric antibody (cPiPP) exhibiting high affinity for hCGβ/hCG and conjugated it to curcumin. It appeared that the antibody by itself does not impair MOLT-4 and U937 cell growth, whereas the novel curcumin conjugate appeared lethal to these cells. Moreover, such curcumin immunoconjugates were shown to kill specifically tumor cells bearing the CD33 marker expressed by acute myeloid leukemia (AML) cells in patients synthetizing and expressing hCGβ on the cellular membrane. Such conjugates do not bind nor affect peripheral blood mononuclear cells (PBMCs) of normal healthy donors. These results underline the fact that conjugation of curcumin to such antibodies improves the transport of this natural molecule to tumor target cells in aqueous medium due to an important increase of its solubility, with a good differential toxicity [126].

### 4.5. Curcumin and Thalidomide

Based on the fact that curcumin overcomes chemoresistance and sensitizes multiple myeloma cells to thalidomide and bortezomib by downregulating NF-κB and NF-κB-regulated genes [127], recent studies focused on the design and biological characterization of hybrid compounds associating curcumin with these two molecules [128]. Five hybrids were designed by taking into account that the phenolic oxygen of curcumin can be modified without significant modification of its biological activity. These hybrids result from the addition of an ester linkage between both compounds, from the replacement of one of the 4-hydroxy-3-methoxy-phenyl rings of curcumin by the phthalimide moiety of thalidomide, from the incorporation of the structural features of both compounds through a benzylidene connection at the methylene position between the carbonyls of curcumin, or from the removal of the 4-hydroxy-3-methoxy-phenyl ring, or from the production of a mono-ketone. These hybrids were tested on MM1S, RPMI18226 and U266 human multiple myeloma (MM) cells to evaluate their impact on cell viability and proliferation. Results showed that the 4-hydroxy-3-methoxy-phenyl ring is essential for the antiproliferative activity of these hybrids, as the two thalidomide hybrids, presented in Figure 4, were the only ones to appear more effective than curcumin alone or combined with thalidomide. Curcumin and these two curcumin-thalidomide-based hybrids generated higher levels of ROS after treatment compared to curcumin alone. This ROS production was shown to trigger cell cycle arrest in the S-phase, leading to subsequent induction of MM cell death by apoptosis. Moreover, these two curcumin hybrids were reported to inhibit tumor necrosis factor alpha (TNFα)-induced activation of NF-κB. In conclusion, these newly-synthetized hybrid compounds exhibit all of the properties of curcumin and thalidomide, but with improved biological activities.

### 4.6. Curcumin-Diaminothiazole Hybrids

Among pharmacologically-active compounds from marine organisms, alkaloids appear as a family of highly-active cytotoxic compounds; however, only a few of them have so far reached the clinical stage, due to their limited supply in nature, their complex structural features and the difficulty of synthetizing them economically. Based on the common presence of highly cytotoxic indole derivatives, including topsentins among these marine alkaloids, different thiazole analogs of topsentin were synthetized. Juneja *et al.* designed diaminoindoloylthiazoles (DIT) and diaminocinnamoylthiazoles (DCT1-2) as novel curcumin-diaminothiazole hybrids. DITs are topsentin analogs in which the indolylimidazole group has been replaced with a 2,4-diaminothiazole unit, whereas DCTs are compounds in which the indoloyl unit in diaminoindoloylthiazole has been substituted with a cinnamoyl group, generating thusly a diarylheptanoid curcumin hybrid (Figure 4). Results showed that both diaminothiazoles inhibited cell growth and induced apoptotic cell death of HeLa human cervical adenocarcinoma cells through intrinsic pathways implicating caspases-3 and -9, by reducing the mitochondrial membrane potential and activating caspases. Even if curcumin-diaminothiazole hybrids appeared active, they were less effective than diaminoindoloylthiazoles (DIT), as they required the highest concentrations to induce a similar level of cell death in HeLa cells by the mitochondrial apoptotic pathway. However, DCT1 appeared as the most effective inhibitor of TNFα-induced NF-κB activation compared to DIT or curcumin alone [129].

### 4.7. Oxovanadium (IV) Curcumin Complexes

Curcumin is mainly described as a chemopreventive or chemotherapeutic compound, but less often presented as a potential photosensitizer. In fact, curcumin could be used in photodynamic therapy (PDT) due to the fact that this fluorescent pigment absorbs around 455 nm and emits between 500 and 578 nm [130,131]. PDT is a treatment strategy based on the administration of photosensitizing drugs (PS), which can, upon illumination, interact with light and intracellular oxygen to generate ROS. This type of cancer therapy shows side effects (e.g., skin photosensitization) that could be abrogated by the use of biocompatible transition-metal complexes, such as ferrocene-conjugated oxovanadium (IV) complexes. Oxovanadium (IV) complexes are also characterized as photoactivatable compounds able to photocleave DNA upon exposure to near-infrared light and to induce cancer cell apoptosis through the intrinsic mitochondrial pathway [132].

The strategy of complexation of curcumin to an oxovanadium (IV) moiety was shown to enhance the photocytotoxicity upon exposition to visible light. The emission property of curcumin can be used to follow the intracellular localization of this complex. Finally, the hydrolytically unstable curcumin can be stabilized by binding to an oxovanadium (IV) moiety [131,133,134].

### 4.8. Binding of Curcumin to β-Lactoglobulin

Binding of curcumin to β-lactoglobulin is linked to the hydrophobic interaction of the polyphenolic rings of curcumin with the hydrophobic pockets of β-lactoglobulin. This leads to an alteration of the β-lactoglobulin conformation with a major reduction of the β-sheet and an increase, in turn, of the structure, causing a partial protein structural destabilization. β-lactoglobulin acts thus as a carrier to transport polyphenols *in vitro* [135]. This type of interaction was shown to increase the bioavailability of curcumin and to improve its antioxidant activity, usually related to its phenolic hydrogen atoms [136].

### 4.9. Limitations of the Hybrid Approach

In some instances, depending on the associated molecules, hybrid molecules do not potentialize the anticancer effect of drugs when applied alone. This was the case when curcumin was conjugated to compounds, such as 3α- and 3β-methoxyserrat-14-en-21β-ol and paclitaxel.

The evaluation of the effect of 3α- and 3β-methoxyserrat-14-en-21β-ol-curcumin conjugates on Epstein-Barr virus early antigen activation (EBV-EA) induced by 12-O-tetradecanoylphorbol-13-acetate (TPA) revealed that such curcumin conjugates exhibited dose-dependent inhibitory activities; however, their cytotoxic potential against Raji cells appeared moderate *in vitro* by comparison with the results obtained for quercetin conjugates designed with the same triterpenoids [137]. Similarly, the conjugation, through an ester linkage, of curcumin to paclitaxel, a natural chemotherapeutic drug already used in the clinic, did not improve either the antioxidant or the cytotoxic properties of paclitaxel [138], whereas its conjugation with camptothecin resulted in a five-fold increase of cytotoxicity against human prostate carcinoma PC-3 cells and in decreased side effects on normal cells compared to the original paclitaxel molecule [139]. Such observations underline the fact that the hybrid molecule concept does not always improve the efficacy of the conjugated molecules.

## 5. Curcumin Hybrids Used for the Treatment of Other Multipotent Diseases

The design of hybrid curcumin molecules was also applied for the discovery of new drugs for the treatment of other multipotent diseases, such as HIV and neurodegenerative disorders.

It appeared that in the case of 3α-methoxyserrat-14-en-21β-ol and 3β-methoxyserrat-14-en-21β-ol-curcumin conjugates, as for their moderate efficacy against cancer cells *in vitro*, these conjugates did not exhibit significant anti-HIV activity compared to kojic acid conjugates, as shown by the evaluation of their impact on anti-HIV-1 reverse transcriptase activity in infected C8166-CCR5 cells, a human CD4+ T-lymphocyte cell line [140].

However, based on the well-described effectiveness, mechanisms of action and limitations of curcumin in neurodegenerative diseases [141,142], the concept of hybrid curcumin molecules was also considered for the treatment of Alzheimer’s disease. AD is a progressive multifactorial neurodegenerative disorder in which several factors, such as aggregation of β-amyloid (Aβ) in the brain or oxidative stress, play important roles in the pathogenesis process. The current symptomatic treatment of AD consists of the use of four acetylcholinesterase inhibitors, such as rivastigmine. Curcumin is also described for its neuroprotective functions, due to its effect on Aβ, and its anti-inflammatory, anti-oxidant and metal chelating properties related to its ortho-methoxy phenol moiety [142,143].

**Figure 5:**
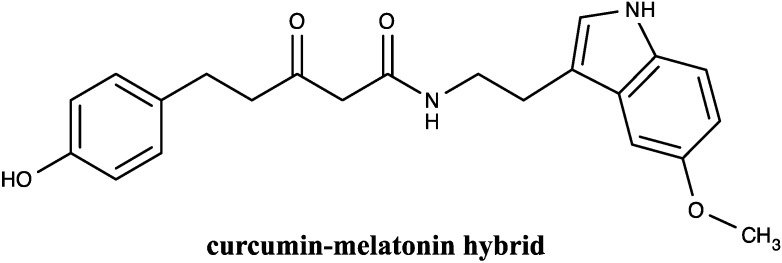
Chemical structure of curcumin hybrid compound applied in Alzheimer’s disease.

In order to improve the treatment of AD, several bivalent multifunctional Aβ oligomerization inhibitors were designed. On the one hand, the ortho-methoxy phenol moiety from curcumin was incorporated in the structure of rivastigmine, a compound exhibiting a modest positive effect on memory and cognitive functions. The resulting hybrid appeared to be a more potent acetylcholinesterase inhibitor than rivastigmine alone, while the Aβ aggregation inhibitory activity was attributed to curcumin [144]. Similarly, multifunctional compounds containing curcumin and cholesterol appeared as potential bivalent multifunctional Aβ oligomerization inhibitors. Moreover, these assays underlined the fact that the length and the attachment position of the spacer that links curcumin and cholesterol are important structural determinants for their biological activities [145]. Another kind of hybrid, 5-(4-hydroxyphenyl)-3-oxo-pentanoic acid [2-(5methoxy-1H-indol-3-yl)-ethyl]-amide, resulting from the association of curcumin and melatonin, was reported to show significant neuroprotective effects that correlate with its antioxidant potential and to its interactions with Aβ oligomers within the mitochondria, but exhibited no effect on Aβ aggregation, at nanomolar concentration ranges in MC65AD cells [146] (Figure 5).

The neuroprotective natural compound, curcumin, could also be combined with cyclohexyl bisphenol A to generate the CNB-001 [4-((1E)-2-(5-(4-hydroxy-3-methoxystyryl-)-1-phenyl-1H-pyrazoyl-3-yl)vinyl)-2-methoxy-phenol)] hybrid molecule [147]. Even CNB-001 decreases the GSH and ATP level; its neuroprotective effect is not related to oxidative stress or mitochondrial toxicity, but to its cytostatic potential. This hybrid compound was also shown to produce some adverse effects at high concentrations. However, it presents significant preclinical efficacy, both *in vitro* and *in vivo*, and appeared thusly as a safe neurotropic and neuroprotective lead compound for the treatment of stroke in a therapeutic safety window of concentration.

In the case of men suffering from diabetes, erectile dysfunction is often observed. Studies performed in diabetes-induced rats pointed out that treatments with water soluble curcumin protein conjugates enhanced erectile function with increased efficiency and prolonged duration of action compared to pure curcumin. This kind of curcumin hybrid led to a significant elevation of intracavernosal pressure (ICP), cyclic guanosine monophosphate (cGMP) hemoxygenase-1 (HO-1) and neuronal NOS (nNOS), as well as a decrease of nuclear transcription factor-erythroid2 (Nrf2), NF-κB, p38 and iNOS [148,149].

Finally, it appeared that curcumin hybrids conjugating curcumin with amino acids not only exhibit antiproliferative potential, but could also present antimicrobial activity [112]. In that case, monoesters of curcumin presented better antimicrobial activity than their corresponding diesters, emphasizing the role of free phenolic groups.

On the other hand, the efficacy of curcumin hybrid molecules in the previously described multipotent diseases could thus provide additional insights into novel molecule dimers to be conjugated and tested for their potential translation into disease treatment.

## 6. Conclusions

The major obstacle for an efficient clinical use of curcumin in the treatment of multifactorial diseases, such as cancer and Alzheimer’s disease, is linked to its low bioavailability. Strategies, such as curcumin nano-formulations, the synthesis of specific analogs or combinations with other components hold some promise. However, the concept of hybrid synthesis appeared the most promising. Unlike the principle of drug combination for cancer treatment, the biological activity of hybrid compounds basically results from a single molecule in which two or more active compounds, with specific mechanisms of action and biological targets, are gathered through covalent chemical bonds. The design of such multifactorial compounds is based on the computation of knowledge about compounds’ SAR, network complexity and signaling pathways implicated in the targeted diseases. Taking into account all of these advances in the field of drug discovery, it has become evident that developing hybrid curcumin compounds will lead to increased *in vivo* bioactivities and could also potentiate the efficacy of conventional chemotherapeutic drugs and overcome the drug resistance process in patients. Some technical challenges will have to be overcome before hybrid drugs succeed in the clinical settings, but the considerable promise of this novel concept generates hope for patients.

## References

[1] DeVita V.T., Chu E. (2008). A history of cancer chemotherapy. Cancer Res..

[2] Kong D.X., Li X.J., Zhang H.Y. (2009). Where is the hope for drug discovery? Let history tell the future. Drug Discov. Today.

[3] Patwardhan B., Mashelkar R.A. (2009). Traditional medicine-inspired approaches to drug discovery: Can ayurveda show the way forward?. Drug Discov. Today.

[4] Rather M.A., Bhat B.A., Qurishi M.A. (2013). Multicomponent phytotherapeutic approach gaining momentum: Is the “one drug to fit all” model breaking down?. Phytomedicine.

[5] Mukherjee P.K., Wahile A. (2006). Integrated approaches towards drug development from ayurveda and other indian system of medicines. J. Ethnopharmacol..

[6] Keith C.T., Borisy A.A., Stockwell B.R. (2005). Multicomponent therapeutics for networked systems. Nat. Rev. Drug Discov..

[7] Dancey J.E., Chen H.X. (2006). Strategies for optimizing combinations of molecularly targeted anticancer agents. Nat. Rev. Drug Discov..

[8] Zimmermann G.R., Lehar J., Keith C.T. (2007). Multi-target therapeutics: When the whole is greater than the sum of the parts. Drug Discov. Today.

[9] Morphy R., Rankovic Z. (2005). Designed multiple ligands. An emerging drug discovery paradigm. J. Med. Chem..

[10] Medina-Franco J.L., Giulianotti M.A., Welmaker G.S., Houghten R.A. (2013). Shifting from the single to the multi-target paradigm in drug discovery. Drug Discov. Today.

[11] Paterson I., Anderson E.A. (2005). Chemistry. The renaissance of natural products as drug candidates. Science.

[12] Bhanot A., Sharma R., Noolvi M.N. (2011). Natural sources as potential anti-cancer agents: A review. Int. J. Phytomed..

[13] Kelkel M., Jacob C., Dicato M., Diederich M. (2010). Potential of the dietary antioxidants resveratrol and curcumin in prevention and treatment of hematologic malignancies. Molecules.

[14] Orlikova B., Diederich M. (2012). Power from the garden: Plant compounds as inhibitors of the hallmarks of cancer. Curr. Med. Chem..

[15] Sawadogo W.R., Schumacher M., Teiten M.H., Cerella C., Dicato M., Diederich M. (2013). A survey of marine natural compounds and their derivatives with anti-cancer activity reported in 2011. Molecules.

[16] Folmer F., Jaspars M., Dicato M., Diederich M. (2008). Marine natural products as targeted modulators of the transcription factor nf-kappab. Biochem. Pharmacol..

[17] Koeberle A., Werz O. (2014). Multi-target approach for natural products in inflammation. Drug Discov. Today.

[18] Aggarwal B.B., Bhatt I.D., Ichikawa H., Ahn K.S., Sethi G., Sandur S.K., Sundaram C., Seeram N., Shishodia S. (2007). Curcumin: Biological and medicinal properties. Turmeric: The Genus Curcuma.

[19] Hatcher H., Planalp R., Cho J., Torti F.M., Torti S.V. (2008). Curcumin: From ancient medicine to current clinical trials. Cell. Mol. Life Sci..

[20] Teiten M.H., Eifes S., Dicato M., Diederich M. (2010). Curcumin-the paradigm of a multi-target natural compound with applications in cancer prevention and treatment. Toxins (Basel).

[21] Teiten M.H., Gaigneaux A., Chateauvieux S., Billing A.M., Planchon S., Fack F., Renaut J., Mack F., Muller C.P., Dicato M. (2012). Identification of differentially expressed proteins in curcumin-treated prostate cancer cell lines. Omics.

[22] Goel A., Kunnumakkara A.B., Aggarwal B.B. (2008). Curcumin as “curecumin”: From kitchen to clinic. Biochem. Pharmacol..

[23] Bar-Sela G., Epelbaum R., Schaffer M. (2010). Curcumin as an anti-cancer agent: Review of the gap between basic and clinical applications. Curr. Med. Chem..

[24] Aggarwal B.B., Kumar A., Bharti A.C. (2003). Anticancer potential of curcumin: Preclinical and clinical studies. Anticancer Res..

[25] Jurenka J.S. (2009). Anti-inflammatory properties of curcumin, a major constituent of curcuma longa: A review of preclinical and clinical research. Altern. Med. Rev..

[26] Epstein J., Sanderson I.R., Macdonald T.T. (2010). Curcumin as a therapeutic agent: The evidence from *in vitro*, animal and human studies. Br. J. Nutr..

[27] Teiten M.H., Dicato M., Diederich M. (2013). Curcumin as a regulator of epigenetic events. Mol. Nutr. Food Res..

[28] Reuter S., Gupta S.C., Park B., Goel A., Aggarwal B.B. (2011). Epigenetic changes induced by curcumin and other natural compounds. Genes Nutr..

[29] Balogun E., Hoque M., Gong P., Killeen E., Green C.J., Foresti R., Alam J., Motterlini R. (2003). Curcumin activates the haem oxygenase-1 gene via regulation of nrf2 and the antioxidant-responsive element. Biochem. J..

[30] Sandur S.K., Pandey M.K., Sung B., Ahn K.S., Murakami A., Sethi G., Limtrakul P., Badmaev V., Aggarwal B.B. (2007). Curcumin, demethoxycurcumin, bisdemethoxycurcumin, tetrahydrocurcumin and turmerones differentially regulate anti-inflammatory and anti-proliferative responses through a ros-independent mechanism. Carcinogenesis.

[31] Menon V.P., Sudheer A.R. (2007). Antioxidant and anti-inflammatory properties of curcumin. Adv. Exp. Med. Biol..

[32] Reuter S., Charlet J., Juncker T., Teiten M.H., Dicato M., Diederich M. (2009). Effect of curcumin on nuclear factor kappab signaling pathways in human chronic myelogenous k562 leukemia cells. Ann. N. Y. Acad. Sci..

[33] Teiten M.H., Eifes S., Reuter S., Duvoix A., Dicato M., Diederich M. (2009). Gene expression profiling related to anti-inflammatory properties of curcumin in k562 leukemia cells. Ann. N. Y. Acad. Sci..

[34] Duvoix A., Blasius R., Delhalle S., Schnekenburger M., Morceau F., Henry E., Dicato M., Diederich M. (2005). Chemopreventive and therapeutic effects of curcumin. Cancer Lett..

[35] Reuter S., Eifes S., Dicato M., Aggarwal B.B., Diederich M. (2008). Modulation of anti-apoptotic and survival pathways by curcumin as a strategy to induce apoptosis in cancer cells. Biochem. Pharmacol..

[36] Bhandarkar S.S., Arbiser J.L. (2007). Curcumin as an inhibitor of angiogenesis. Adv. Exp. Med. Biol..

[37] Kunnumakkara A.B., Anand P., Aggarwal B.B. (2008). Curcumin inhibits proliferation, invasion, angiogenesis and metastasis of different cancers through interaction with multiple cell signaling proteins. Cancer Lett..

[38] Reddy A.R., Dinesh P., Prabhakar A.S., Umasankar K., Shireesha B., Raju M.B. (2013). A comprehensive review on sar of curcumin. Mini Rev. Med. Chem..

[39] Priyadarsini K.I. (2013). Chemical and structural features influencing the biological activity of curcumin. Curr. Pharm. Des..

[40] Jitoe-Masuda A., Fujimoto A., Masuda T. (2013). Curcumin: From chemistry to chemistry-based functions. Curr. Pharm. Des..

[41] Fuchs J.R., Pandit B., Bhasin D., Etter J.P., Regan N., Abdelhamid D., Li C., Lin J., Li P.K. (2009). Structure-activity relationship studies of curcumin analogues. Bioorg. Med. Chem. Lett..

[42] Agrawal R., Mishra B., Tyagi E., Nath C., Shukla R. (2010). Effect of curcumin on brain insulin receptors and memory functions in stz (icv) induced dementia model of rat. Pharmacol. Res..

[43] Ishida J., Ohtsu H., Tachibana Y., Nakanishi Y., Bastow K.F., Nagai M., Wang H.K., Itokawa H., Lee K.H. (2002). Antitumor agents. Part 214: Synthesis and evaluation of curcumin analogues as cytotoxic agents. Bioorg. Med. Chem..

[44] Sugiyama Y., Kawakishi S., Osawa T. (1996). Involvement of the beta-diketone moiety in the antioxidative mechanism of tetrahydrocurcumin. Biochem. Pharmacol..

[45] Somparn P., Phisalaphong C., Nakornchai S., Unchern S., Morales N.P. (2007). Comparative antioxidant activities of curcumin and its demethoxy and hydrogenated derivatives. Biol. Pharm. Bull..

[46] Ferrari E., Lazzari S., Marverti G., Pignedoli F., Spagnolo F., Saladini M. (2009). Synthesis, cytotoxic and combined cddp activity of new stable curcumin derivatives. Bioorg. Med. Chem..

[47] Lopez-Lazaro M. (2008). Anticancer and carcinogenic properties of curcumin: Considerations for its clinical development as a cancer chemopreventive and chemotherapeutic agent. Mol. Nutr. Food Res..

[48] Burgos-Moron E., Calderon-Montano J.M., Salvador J., Robles A., Lopez-Lazaro M. (2010). The dark side of curcumin. Int. J. Cancer.

[49] Anand P., Kunnumakkara A.B., Newman R.A., Aggarwal B.B. (2007). Bioavailability of curcumin: Problems and promises. Mol. Pharm..

[50] Bisht S., Maitra A. (2009). Systemic delivery of curcumin: 21st century solutions for an ancient conundrum. Curr. Drug Discov. Technol..

[51] Shehzad A., Khan S., Shehzad O., Lee Y.S. (2010). Curcumin therapeutic promises and bioavailability in colorectal cancer. Drugs Today (Barc.).

[52] Liu Z., Tang L., Zou P., Zhang Y., Wang Z., Fang Q., Jiang L., Chen G., Xu Z., Zhang H. (2014). Synthesis and biological evaluation of allylated and prenylated mono-carbonyl analogs of curcumin as anti-inflammatory agents. Eur. J. Med. Chem..

[53] Tamvakopoulos C., Dimas K., Sofianos Z.D., Hatziantoniou S., Han Z., Liu Z.L., Wyche J.H., Pantazis P. (2007). Metabolism and anticancer activity of the curcumin analogue, dimethoxycurcumin. Clin. Cancer Res..

[54] Steward W.P., Gescher A.J. (2008). Curcumin in cancer management: Recent results of analogue design and clinical studies and desirable future research. Mol. Nutr. Food Res..

[55] Patwardhan R.S., Checker R., Sharma D., Kohli V., Priyadarsini K.I., Sandur S.K. (2011). Dimethoxycurcumin, a metabolically stable analogue of curcumin, exhibits anti-inflammatory activities in murine and human lymphocytes. Biochem. Pharmacol..

[56] Ohori H., Yamakoshi H., Tomizawa M., Shibuya M., Kakudo Y., Takahashi A., Takahashi S., Kato S., Suzuki T., Ishioka C. (2006). Synthesis and biological analysis of new curcumin analogues bearing an enhanced potential for the medicinal treatment of cancer. Mol. Cancer Ther..

[57] Yadav B., Taurin S., Rosengren R.J., Schumacher M., Diederich M., Somers-Edgar T.J., Larsen L. (2010). Synthesis and cytotoxic potential of heterocyclic cyclohexanone analogues of curcumin. Bioorg. Med. Chem..

[58] Kasinski A.L., Du Y., Thomas S.L., Zhao J., Sun S.Y., Khuri F.R., Wang C.Y., Shoji M., Sun A., Snyder J.P. (2008). Inhibition of ikappab kinase-nuclear factor-kappab signaling pathway by 3,5-bis(2-flurobenzylidene)piperidin-4-one (ef24), a novel monoketone analog of curcumin. Mol. Pharmacol..

[59] Zambre A.P., Kulkarni V.M., Padhye S., Sandur S.K., Aggarwal B.B. (2006). Novel curcumin analogs targeting tnf-induced nf-kappab activation and proliferation in human leukemic kbm-5 cells. Bioorg. Med. Chem..

[60] Meghwal M., Goswami T.K. (2013). Piper nigrum and piperine: An update. Phytother. Res..

[61] Srinivasan K. (2007). Black pepper and its pungent principle-piperine: A review of diverse physiological effects. Crit Rev. Food Sci. Nutr..

[62] Shoba G., Joy D., Joseph T., Majeed M., Rajendran R., Srinivas P.S. (1998). Influence of piperine on the pharmacokinetics of curcumin in animals and human volunteers. Planta Med..

[63] Kondo A., Takeda T., Li B., Tsuiji K., Kitamura M., Wong T.F., Yaegashi N. (2013). Epigallocatechin-3-gallate potentiates curcumin’s ability to suppress uterine leiomyosarcoma cell growth and induce apoptosis. Int. J. Clin. Oncol..

[64] Naksuriya O., Okonogi S., Schiffelers R.M., Hennink W.E. (2014). Curcumin nano-formulations: A review of pharmaceutical properties and preclinical studies and clinical data related to cancer treatment. Biomaterials.

[65] Shehzad A., Ul-Islam M., Wahid F., Lee Y.S. (2014). Multifunctional polymeric nanocurcumin for cancer therapy. J. Nanosci. Nanotechnol..

[66] Yallapu M.M., Jaggi M., Chauhan S.C. (2012). Curcumin nano-formulations: A future nanomedicine for cancer. Drug Discov. Today.

[67] Yang R., Zhang S., Kong D., Gao X., Zhao Y., Wang Z. (2012). Biodegradable polymer-curcumin conjugate micelles enhance the loading and delivery of low-potency curcumin. Pharm. Res..

[68] Mohanty C., Acharya S., Mohanty A.K., Dilnawaz F., Sahoo S.K. (2010). Curcumin-encapsulated mepeg/pcl diblock copolymeric micelles: A novel controlled delivery vehicle for cancer therapy. Nanomedicine (Lond.).

[69] Sahu A., Bora U., Kasoju N., Goswami P. (2008). Synthesis of novel biodegradable and self-assembling methoxy poly(ethylene glycol)-palmitate nanocarrier for curcumin delivery to cancer cells. Acta Biomater..

[70] Gou M., Men K., Shi H., Xiang M., Zhang J., Song J., Long J., Wan Y., Luo F., Zhao X. (2011). Curcumin-loaded biodegradable polymeric micelles for colon cancer therapy *in vitro* and *in vivo*. Nanoscale.

[71] Dey S., Sreenivasan K. (2014). Conjugation of curcumin onto alginate enhances aqueous solubility and stability of curcumin. Carbohydr. Polym..

[72] Manju S., Sreenivasan K. (2011). Conjugation of curcumin onto hyaluronic acid enhances its aqueous solubility and stability. J. Colloid Interface Sci..

[73] Goyal P., Goyal K., Vijaya Kumar S.G., Singh A., Katare O.P., Mishra D.N. (2005). Liposomal drug delivery systems--clinical applications. Acta Pharm..

[74] Li L., Braiteh F.S., Kurzrock R. (2005). Liposome-encapsulated curcumin: *In vitro* and *in vivo* effects on proliferation, apoptosis, signaling, and angiogenesis. Cancer.

[75] Hasan M., Belhaj N., Benachour H., Barberi-Heyob M., Kahn C.J., Jabbari E., Linder M., Arab-Tehrany E. (2014). Liposome encapsulation of curcumin: Physico-chemical characterizations and effects on mcf7 cancer cell proliferation. Int. J. Pharm..

[76] Saengkrit N., Saesoo S., Srinuanchai W., Phunpee S., Ruktanonchai U.R. (2014). Influence of curcumin-loaded cationic liposome on anticancer activity for cervical cancer therapy. Colloids Surf. B Biointerfaces.

[77] Wang D., Veena M.S., Stevenson K., Tang C., Ho B., Suh J.D., Duarte V.M., Faull K.F., Mehta K., Srivatsan E.S. (2008). Liposome-encapsulated curcumin suppresses growth of head and neck squamous cell carcinoma *in vitro* and in xenografts through the inhibition of nuclear factor kappab by an akt-independent pathway. Clin. Cancer Res..

[78] Li L., Ahmed B., Mehta K., Kurzrock R. (2007). Liposomal curcumin with and without oxaliplatin: Effects on cell growth, apoptosis, and angiogenesis in colorectal cancer. Mol. Cancer Ther..

[79] Narayanan N.K., Nargi D., Randolph C., Narayanan B.A. (2009). Liposome encapsulation of curcumin and resveratrol in combination reduces prostate cancer incidence in pten knockout mice. Int. J. Cancer.

[80] Singh A., Dilnawaz F., Mewar S., Sharma U., Jagannathan N.R., Sahoo S.K. (2011). Composite polymeric magnetic nanoparticles for co-delivery of hydrophobic and hydrophilic anticancer drugs and mri imaging for cancer therapy. ACS Appl. Mater. Interfaces.

[81] Balasubramanian S., Girija A.R., Nagaoka Y., Iwai S., Suzuki M., Kizhikkilot V., Yoshida Y., Maekawa T., Nair S.D. (2014). Curcumin and 5-fluorouracil-loaded, folate- and transferrin-decorated polymeric magnetic nano-formulation: A synergistic cancer therapeutic approach, accelerated by magnetic hyperthermia. Int. J. Nanomed..

[82] Pramanik D., Campbell N.R., Das S., Gupta S., Chenna V., Bisht S., Sysa-Shah P., Bedja D., Karikari C., Steenbergen C. (2012). A composite polymer nanoparticle overcomes multidrug resistance and ameliorates doxorubicin-associated cardiomyopathy. Oncotarget.

[83] Misra R., Sahoo S.K. (2011). Coformulation of doxorubicin and curcumin in poly(d,l-lactide-co-glycolide) nanoparticles suppresses the development of multidrug resistance in k562 cells. Mol. Pharm..

[84] Frei E., Eder J.P., Kufe D.W., Weichselbaum R.R., Bast R.C., Gansler T.S., Holland J.F., Frei E. (2003). Combination chemotherapy. Holland-Frei Cancer Medicine.

[85] Meunier B. (2008). Hybrid molecules with a dual mode of action: Dream or reality?. Acc. Chem. Res..

[86] Chauhan S.S., Sharma M., Chauhan P.M. (2010). Trioxaquines: Hybrid molecules for the treatment of malaria. Drug News Perspect..

[87] Bajorath J. (2002). Chemoinformatics methods for systematic comparison of molecules from natural and synthetic sources and design of hybrid libraries. J. Comput. Aided Mol. Des..

[88] Jia J., Zhu F., Ma X., Cao Z., Li Y., Chen Y.Z. (2009). Mechanisms of drug combinations: Interaction and network perspectives. Nat. Rev. Drug Discov..

[89] Fortin S., Berube G. (2013). Advances in the development of hybrid anticancer drugs. Expert Opin. Drug Discov..

[90] Mehta G., Singh V. (2002). Hybrid systems through natural product leads: An approach towards new molecular entities. Chem. Soc. Rev..

[91] Nepali K., Sharma S., Kumar D., Budhiraja A., Dhar K.L. (2014). Anticancer hybrids—A patent survey. Recent Pat. Anticancer Drug Discov..

[92] Bansal Y., Silakari O. (2014). Multifunctional compounds: Smart molecules for multifactorial diseases. Eur. J. Med. Chem..

[93] Morphy R., Kay C., Rankovic Z. (2004). From magic bullets to designed multiple ligands. Drug Discov. Today.

[94] Nepali K., Sharma S., Sharma M., Bedi P.M., Dhar K.L. (2014). Rational approaches, design strategies, structure activity relationship and mechanistic insights for anticancer hybrids. Eur. J. Med. Chem..

[95] Muregi F.W., Ishih A. (2010). Next-generation antimalarial drugs: Hybrid molecules as a new strategy in drug design. Drug Dev. Res..

[96] Tietze L.F., Bell H.P., Chandrasekhar S. (2003). Natural product hybrids as new leads for drug discovery. Angew. Chem. Int. Ed..

[97] Gademann K. (2006). Natural produc hybrids. Chimia.

[98] Tsogoeva S.B. (2010). Recent progress in the development of synthetic hybrids of natural or unnatural bioactive compounds for medicinal chemistry. Mini. Rev. Med. Chem..

[99] Decker M. (2011). Hybrid molecules incorporating natural products: Applications in cancer therapy, neurodegenerative disorders and beyond. Curr. Med. Chem..

[100] Li S.Y., Wang X.B., Xie S.S., Jiang N., Wang K.D., Yao H.Q., Sun H.B., Kong L.Y. (2013). Multifunctional tacrine-flavonoid hybrids with cholinergic, beta-amyloid-reducing, and metal chelating properties for the treatment of alzheimer’s disease. Eur. J. Med. Chem..

[101] Mizuno C.S., Paul S., Suh N., Rimando A.M. (2010). Synthesis and biological evaluation of retinoid-chalcones as inhibitors of colon cancer cell growth. Bioorg. Med. Chem. Lett..

[102] Mao F., Yan J., Li J., Jia X., Miao H., Sun Y., Huang L., Li X. (2014). New multi-target-directed small molecules against alzheimer’s disease: A combination of resveratrol and clioquinol. Org. Biomol. Chem..

[103] Belluti F., Fontana G., dal Bo L., Carenini N., Giommarelli C., Zunino F. (2010). Design, synthesis and anticancer activities of stilbene-coumarin hybrid compounds: Identification of novel proapoptotic agents. Bioorg. Med. Chem..

[104] Singh N., Sarkar J., Sashidhara K.V., Ali S., Sinha S. (2014). Anti-tumour activity of a novel coumarin-chalcone hybrid is mediated through intrinsic apoptotic pathway by inducing puma and altering bax/bcl-2 ratio. Apoptosis.

[105] Abdel-Aziz M., Park S.E., Abuo-Rahma Gel D., Sayed M.A., Kwon Y. (2013). Novel n-4-piperazinyl-ciprofloxacin-chalcone hybrids: Synthesis, physicochemical properties, anticancer and topoisomerase i and ii inhibitory activity. Eur. J. Med. Chem..

[106] Wu J.H., Batist G., Zhou J., Geng G., Lin R., Li Y. (2013). Hybrid-Ionone and Curcumin Molecules as Anticancer Agents. U.S. Patent.

[107] Dimauro T.M. (2013). Methylated Curcumin-Resveratrol Hybrid Molecules for Treating Cancer. U.S. Patent.

[108] Dimauro T.M. (2014). Curcumin-Resveratrol Hybrids. European Patent.

[109] Mishra S., Kapoor N., Mubarak Ali A., Pardhasaradhi B.V., Kumari A.L., Khar A., Misra K. (2005). Differential apoptotic and redox regulatory activities of curcumin and its derivatives. Free Radic. Biol. Med..

[110] Mantovani F., Banks L. (2001). The human papillomavirus e6 protein and its contribution to malignant progression. Oncogene.

[111] Singh A.K., Misra K. (2013). Human papilloma virus 16 e6 protein as a target for curcuminoids, curcumin conjugates and congeners for chemoprevention of oral and cervical cancers. Interdiscip. Sci..

[112] Dubey S.K., Sharma A.K., Narain U., Misra K., Pati U. (2008). Design, synthesis and characterization of some bioactive conjugates of curcumin with glycine, glutamic acid, valine and demethylenated piperic acid and study of their antimicrobial and antiproliferative properties. Eur. J. Med. Chem..

[113] Singh D.V., Agarwal S., Singh P., Godbole M.M., Misra K. (2013). Curcumin conjugates induce apoptosis via a mitochondrion dependent pathway in mcf-7 and mda-mb-231 cell lines. Asian Pac. J. Cancer Prev..

[114] Trecul A., Morceau F., Dicato M., Diederich M. (2012). Dietary compounds as potent inhibitors of the signal transducers and activators of transcription (stat) 3 regulatory network. Genes Nutr..

[115] Kumar A., Bora U. (2012). Molecular docking studies on inhibition of stat3 dimerization by curcumin natural derivatives and its conjugates with amino acids. Bioinformation.

[116] Aguilar-Rojas A., Huerta-Reyes M. (2009). Human gonadotropin-releasing hormone receptor-activated cellular functions and signaling pathways in extra-pituitary tissues and cancer cells (review). Oncol. Rep..

[117] Limonta P., Montagnani Marelli M., Mai S., Motta M., Martini L., Moretti R.M. (2012). Gnrh receptors in cancer: From cell biology to novel targeted therapeutic strategies. Endocr. Rev..

[118] Montagnani Marelli M., Moretti R.M., Januszkiewicz-Caulier J., Motta M., Limonta P. (2006). Gonadotropin-releasing hormone (gnrh) receptors in tumors: A new rationale for the therapeutical application of gnrh analogs in cancer patients?. Curr. Cancer Drug Targets.

[119] Grundker C., Gunthert A.R., Westphalen S., Emons G. (2002). Biology of the gonadotropin-releasing hormone system in gynecological cancers. Eur. J. Endocrinol..

[120] Aggarwal S., Ndinguri M.W., Solipuram R., Wakamatsu N., Hammer R.P., Ingram D., Hansel W. (2011). [dlys(6)]-luteinizing hormone releasing hormone-curcumin conjugate inhibits pancreatic cancer cell growth *in vitro* and *in vivo*. Int. J. Cancer.

[121] Shi Q., Wada K., Ohkoshi E., Lin L., Huang R., Morris-Natschke S.L., Goto M., Lee K.H. (2012). Antitumor agents 290. Design, synthesis, and biological evaluation of new lncap and pc-3 cytotoxic curcumin analogs conjugated with anti-androgens. Bioorg. Med. Chem..

[122] Souriau C., Hudson P.J. (2003). Recombinant antibodies for cancer diagnosis and therapy. Expert Opin. Biol. Ther..

[123] List T., Neri D. (2013). Immunocytokines: A review of molecules in clinical development for cancer therapy. Clin. Pharmacol..

[124] Xin L., Cao J., Cheng H., Zeng F., Hu X., Shao J. (2013). Human monoclonal antibodies in cancer therapy: A review of recent developments. Front Biosci. (Landmark Ed.).

[125] Cole L.A. (2012). Hcg variants, the growth factors which drive human malignancies. Am. J. Cancer Res..

[126] Vyas H.K., Pal R., Vishwakarma R., Lohiya N.K., Talwar G.P. (2009). Selective killing of leukemia and lymphoma cells ectopically expressing hcgbeta by a conjugate of curcumin with an antibody against hcgbeta subunit. Oncology.

[127] Sung B., Kunnumakkara A.B., Sethi G., Anand P., Guha S., Aggarwal B.B. (2009). Curcumin circumvents chemoresistance *in vitro* and potentiates the effect of thalidomide and bortezomib against human multiple myeloma in nude mice model. Mol. Cancer Ther..

[128] Liu K., Zhang D., Chojnacki J., Du Y., Fu H., Grant S., Zhang S. (2013). Design and biological characterization of hybrid compounds of curcumin and thalidomide for multiple myeloma. Org. Biomol. Chem..

[129] Juneja M., Vanam U., Paranthaman S., Bharathan A., Keerthi V.S., Reena J.K., Rajaram R., Rajasekharan K.N., Karunagaran D. (2013). 4-amino-2-arylamino-5-indoloyl/cinnamoythiazoles, analogs of topsentin-class of marine alkaloids, induce apoptosis in hela cells. Eur. J. Med. Chem..

[130] Koon H., Leung A.W., Yue K.K., Mak N.K. (2006). Photodynamic effect of curcumin on npc/cne2 cells. J. Environ. Pathol. Toxicol. Oncol..

[131] Banerjee S., Prasad P., Hussain A., Khan I., Kondaiah P., Chakravarty A.R. (2012). Remarkable photocytotoxicity of curcumin in hela cells in visible light and arresting its degradation on oxovanadium(iv) complex formation. Chem. Commun..

[132] Prasad P., Khan I., Kondaiah P., Chakravarty A.R. (2013). Mitochondria-targeting oxidovanadium(iv) complex as a near-ir light photocytotoxic agent. Chemistry.

[133] Banik B., Somyajit K., Nagaraju G., Chakravarty A.R. (2014). Oxovanadium(iv) complexes of curcumin for cellular imaging and mitochondria targeted photocytotoxicity. Dalton Trans..

[134] Balaji B., Somyajit K., Banik B., Nagaraju G., Chakravarty A.R. (2013). Photoactivated DNA cleavage and anticancer activity of oxovanadium(iv) complexes of curcumin. Inorg. Chim. Acta.

[135] Kanakis C.D., Tarantilis P.A., Polissiou M.G., Tajmir-Riahi H.A. (2013). Probing the binding sites of resveratrol, genistein, and curcumin with milk beta-lactoglobulin. J. Biomol. Struct. Dyn..

[136] Li M., Ma Y., Ngadi M.O. (2013). Binding of curcumin to beta-lactoglobulin and its effect on antioxidant characteristics of curcumin. Food Chem..

[137] Tsujii H., Yamada T., Kajimoto T., Tanaka R., Tokuda H., Hasegawa J., Hamashima Y., Node M. (2010). Hybrids of 3alpha-methoxyserrat-14-en-21beta-ol (pj-1) and 3beta-methoxyserrat-14-en-21beta-ol (pj-2) and various anti-oxidants as cancer chemopreventive agents. Eur. J. Med. Chem..

[138] Nakagawa-Goto K., Yamada K., Nakamura S., Chen T.H., Chiang P.C., Bastow K.F., Wang S.C., Spohn B., Hung M.C., Lee F.Y. (2007). Antitumor agents. 258. Syntheses and evaluation of dietary antioxidant—Taxoid conjugates as novel cytotoxic agents. Bioorg. Med. Chem. Lett..

[139] Nakagawa-Goto K., Nakamura S., Bastow K.F., Nyarko A., Peng C.Y., Lee F.Y., Lee F.C., Lee K.H. (2007). Antitumor agents. 256. Conjugation of paclitaxel with other antitumor agents: Evaluation of novel conjugates as cytotoxic agents. Bioorg. Med. Chem. Lett..

[140] Tanaka R., Tsujii H., Yamada T., Kajimoto T., Amano F., Hasegawa J., Hamashima Y., Node M., Katoh K., Takebe Y. (2009). Novel 3alpha-methoxyserrat-14-en-21beta-ol (pj-1) and 3beta-methoxyserrat-14-en-21beta-ol (pj-2)-curcumin, kojic acid, quercetin, and baicalein conjugates as hiv agents. Bioorg. Med. Chem..

[141] Lee W.H., Loo C.Y., Bebawy M., Luk F., Mason R.S., Rohanizadeh R. (2013). Curcumin and its derivatives: Their application in neuropharmacology and neuroscience in the 21st century. Curr. Neuropharmacol..

[142] Chin D., Huebbe P., Pallauf K., Rimbach G. (2013). Neuroprotective properties of curcumin in Alzheimer’s disease--merits and limitations. Curr. Med. Chem..

[143] Reinke A.A., Gestwicki J.E. (2007). Structure-activity relationships of amyloid beta-aggregation inhibitors based on curcumin: Influence of linker length and flexibility. Chem. Biol. Drug Des..

[144] Li Y., Peng P., Tang L., Hu Y., Sheng R. (2014). Design, synthesis and evaluation of rivastigmine and curcumin hybrids as site-activated multi-target-directed ligands for Alzheimer’s disease therapy. Bioorg. Med. Chem..

[145] Lenhart J.A., Ling X., Gandhi R., Guo T.L., Gerk P.M., Brunzell D.H., Zhang S. (2010). “Clicked” bivalent ligands containing curcumin and cholesterol as multifunctional abeta oligomerization inhibitors: Design, synthesis, and biological characterization. J. Med. Chem..

[146] Chojnacki J.E., Liu K., Yan X., Toldo S., Selden T., Estrada M., Rodriguez-Franco M.I., Halquist M.S., Ye D., Zhang S. (2014). Discovery of 5-(4-hydroxyphenyl)-3-oxo-pentanoic acid [2-(5-methoxy-1h-indol-3-yl)-ethyl]-amide as a neuroprotectant for alzheimer’s disease by hybridization of curcumin and melatonin. ACS Chem. Neurosci..

[147] Lapchak P.A., McKim J.M. (2011). Ceetox analysis of cnb-001 a novel curcumin-based neurotrophic/neuroprotective lead compound to treat stroke: Comparison with nxy-059 and radicut. Transl. Stroke Res..

[148] Abdel Aziz M.T., Motawi T., Rezq A., Mostafa T., Fouad H.H., Ahmed H.H., Rashed L., Sabry D., Senbel A., Al-Malki A. (2012). Effects of a water-soluble curcumin protein conjugate *vs.* Pure curcumin in a diabetic model of erectile dysfunction. J. Sex. Med..

[149] Zaahkouk A.M., Abdel Aziz M.T., Rezq A.M., Atta H.M., Fouad H.H., Ahmed H.H., Sabry D., Yehia M.H. (2014). Efficacy of a novel water-soluble curcumin derivative *versus* sildenafil citrate in mediating erectile function. Int. J. Impot. Res..

